# Galectinology of Equine Pregnancy

**DOI:** 10.3390/ani13010129

**Published:** 2022-12-29

**Authors:** Carleigh E. Fedorka, Hossam El-Sheikh Ali, Mats H. T. Troedsson

**Affiliations:** 1 Department of Veterinary Science, University of Kentucky, Lexington, KY 40546-0099, USA; 2 College of Veterinary Medicine, Mansoura University, Mansoura 35516, Egypt

**Keywords:** galectin, equine, pregnancy, chorioallantois, endometrium

## Abstract

**Simple Summary:**

Galectins are a family of proteins that have been implicated in reproduction, including pregnancy maintenance. Unfortunately, minimal is known about galectins in the horse. Therefore, we aimed to evaluate the expression of various galectins throughout equine pregnancy. Chorioallantois (placenta) and endometrium (uterus) were obtained from both nonpregnant and pregnant mares, as well as postpartum. Next-generation sequencing was performed on tissues, and comparisons were made between gestational lengths. In the endometrium, galectin-1 and galectin-13 were found in the highest expression in the nonpregnant mare, with lower expression levels in the pregnant mare. In contrast, galectin-8 and galectin-12 were found to be lowest in the nonpregnant mare and reached the highest expression levels in mid-gestation before declining as parturition neared. In the chorioallantois, galectin-1, galectin-3, and galectin-3BP were found to have heightened expression levels at 45 d of gestation, with lesser expression levels noted throughout gestation. In contrast, galectin-9, galectin-12, and galectin-13 experienced the highest expression levels in late-term pregnancy, with lesser expression noted in early pregnancy. In conclusion, galectins appear to be involved in pregnancy in the horse, and this is dependent on the tissue within the reproductive tract and the specific galectin involved.

**Abstract:**

Galectins are a family of proteins that bind to glycans, acting in a cytokine-like manner throughout the body. In the majority of mammalians, galectins have been found to be involved in pregnancy maintenance, but few studies have evaluated this in the horse. Therefore, the objective of this study was to examine the expression of various galectins in pregnant and nonpregnant mares. Next-generation RNA sequencing was performed on the chorioallantois and endometrium of healthy pregnant mares at 120, 180, 300, and 330 days of gestation (n = 4/stage), as well as 45-day chorioallantois (n = 4), postpartum chorioallantois (n = 3), and diestrus endometrium (n = 3). In the endometrium, galectin-1 and galectin-13 were found in the highest expression in the nonpregnant mare, with decreasing levels of expression noted throughout gestation. In contrast, galectin-8 and galectin-12 were found to be the lowest in the nonpregnant mare and reached the highest expression levels in mid-gestation before declining as parturition neared. In the chorioallantois, galectin-1, galectin-3, and galectin-3BP were found to have heightened expression levels at 45 d of gestation, with lesser expression levels noted throughout gestation. In contrast, galectin-9, galectin-12, and galectin-13 experienced the highest expression levels in the late-term chorioallantois (300 d/330 d), with lesser expression noted in early- to mid-gestation. Of note, galectin-1, galectin-3BP, galectin-9, galectin-12, and galectin-13 all experienced the lowest expression levels in the postpartum placenta, with heightened expression noted during gestation. In conclusion, galectins appear to be involved in equine pregnancy, and this is dependent on both the tissue within the feto-maternal interface and the specific galectin involved.

## 1. Introduction

Galectins are a family of endogenous lectins that are characterized by the presence of a conserved carbohydrate recognition domain (CRD) that binds to glycans in targeted cells. Galectins are found both intra- and extra-cellularly and display a variety of functions, many of which are galectin-specific, including cell–cell interactions, cell proliferation, cell differentiation, cell transformation, and apoptosis. Intracellularly, they engage protein–protein interactions and modulate cell growth, differentiation, migration, and survival [1]. Galectins are also able to be exported from cells by non-classical secretion [2] and function in a cytokine-like manner due to their ability to influence cell adhesion, apoptosis, and the regulation of both the innate and adaptive immune systems [3,4].

In the human, members of the galectin family have been found to be involved in numerous reproductive processes, including the maternal immunotolerance of the semi-allogeneic fetus, implantation, placentation, and endocrine–immune interactions. This includes, but is not limited to, a stimulation of regulatory T cell production [5], remodeling of the extracellular matrix at the feto-maternal interface [6,7], and the rescue of a progesterone-deprived pregnancy [8]. Therefore, it is believed that galectins drive the successful establishment and maintenance of pregnancy. It should be noted that, among the various galectins that have been described in the literature, many are species-specific, such as galectin-5 in mice [9], galectin-11 in ruminants [10], and galectin-15 in sheep and goats [11]. To date, minimal work has gone into the study of galectins in the horse or their involvement in equine pregnancy.

The implantation, placentation, and endocrinology of equine pregnancy differ from those of human pregnancy, and, therefore, inferences are difficult to make. In contrast to the human, the equine pregnancy is only regulated by ovarian-produced progesterone for the first 120 days, after which the progestins 5α-DHP and 20α-5P are produced by the feto-placental unit [12]. Additionally, a true implantation event does not occur until the formation of the gonadotropin-secreting endometrial cups, which develop at roughly day 35 of gestation and degrade between 80 and 120 days [13,14]. In addition, the equine pregnancy requires a dynamic adaptive immune response throughout gestation and is governed by a suppressed Th1, enhanced Th2, and fluctuating Treg response at the feto-maternal interface [15]. Interestingly, galectin-1 was found to be immunolocalized on trophoblast cells in equine pregnancy [16]. To our knowledge, no other galectin has been investigated during equine gestation, and none have been investigated outside of early pregnancy. 

Therefore, additional information is necessary to determine the involvement of galectins in pregnancy maintenance in the horse. The epitheliochorial placentation of the horse allows for increased specificity when examining the dynamics of the feto-maternal interface [17]. We hypothesize that the expression of individual galectins will differ in the non-pregnant mares and throughout gestation due to their varying downstream effects. Therefore, the objective of this study was to examine the expression of various galectins in the chorioallantois and endometrium of the pregnant mare at varying gestational lengths. It is essential that we understand the normal equine pregnancy, as any imbalance in any of these outcomes may increase the risk of pregnancy loss and can therefore be used as a marker for fetal viability and pregnancy complications or precede therapeutic intervention.

## 2. Material and Methods

### 2.1. Animal Use and Tissue Collection

All animal procedures were approved by and completed in accordance with the Institutional Animal Care and Use Committee of the University of Kentucky (Protocols #2014-1215 and 2014-1341). All horses (*Equus caballus*) used in this study were mares of mixed breed and parity ranging from 250 to 600 kg and from four to sixteen years of age. Mares were housed on pasture, with free-choice grass hay available at all times. Mares were bred via pasture breeding to two stallions of known fertility and pregnancy, as confirmed by transrectal ultrasonography, between 14 and 35 days of gestation [18]. Both chorioallantois (CA) and endometrium (EN) were collected at a range of gestational ages, including 45 d (CA only; n = 4), 120 d (CA and EN; n = 4), 180 d (CA and EN; n = 4), 300 d (CA and EN; n = 4), 330 d (CA and EN; n = 4), and diestrus (7 days post-ovulation, EN only; n = 3). Forty-five-day CA was obtained from embryos collected via uterine lavage with 2 L LRS, using a 10 cm tracheal tube inserted through the cervix. Intrauterine infusion with LRS was followed by treatment with 10 U of oxytocin IV to assist with expelling the intact embryo. The chorionic girdle was identified from the expelled embryo, and trophoblastic cells were collected accordingly. All other tissues were collected post-mortem from the mare following euthanasia by pentobarbitol, as described by El-Sheikh Ali et al. (2021) [19]. CA and EN were carefully dissected from surrounding tissues and then stored in either RNAlater (Thermo Fisher Scientific, Waltham, MA, USA) or neutral buffered formalin (*w*/*v*) for 24 h followed by methanol for immunohistochemistry (IHC). 

### 2.2. RNA Isolation

The isolation of RNA from chorioallantois and endometrium was performed using an RNeasy Mini Kit (Qiagen, Gaithersburg, MD, USA), per the manufacturer’s instructions and as previously described by El-Sheikh Ali et al. [20]. RNA concentration, purity, and integrity were analyzed by Bioanalyzer^®^ (Agilent, Santa Clara, CA, USA). All samples had a 230/260 ratio > 1.8, a 260/280 ratio > 2.0, and an RNA integrity number > 8.0. Library preparation was performed using the TruSeq Stranded mRNA Sample Prep Kit (Illumina), as per the manufacturer’s instructions. All reads were quantified with qPCR. 

### 2.3. RNA Sequencing

Next-generation RNA-seq was carried out on both chorioallantois and endometrium. Briefly, sequencing libraries were generated using the NEBNext^®^ Ultra™ RNA Library Prep Kit for Illumina^®^ (gSan Diego, CA, USA), following the manufacturer’s recommendations. Library preparations were sequenced on NovaSeq 6000, and 125 bp/150 bp paired-end reads were generated. The reads were then trimmed for quality and adapters with TrimGalore 0.4.3 and then mapped to EquCab3.0 using hisat2 v2.1 [21]. The expression values (fragments per kilobase measured; FPKM) of the mapped reads were quantified using FeatureCounts for v1.5.0 [22], with the NCBI annotation (GCF_002863925.1). The expression of galectin-1, galectin-2, galectin-3, galectin-3BP, galectin-4, galectin-5, galectin-6, galectin-7, galectin-8, galectin-9, galectin-9B, galectin-10, galectin-11, galectin-12, galectin-13, galectin-14, galectin-15, galectin-L, and GRIFIN was assessed.

### 2.4. Statistics

All statistical analyses were performed in SAS 9.4 (SAS Institute, version 12.1.0). Statistical comparisons of FPKM were performed using a general linear model, with post-hoc analysis by Tukey’s HSD. Tissues were compared across gestational ages. Descriptive statistics are expressed as the mean ± SE unless otherwise stated. Significance was set to *p* ≤ 0.05, with a trend noted at *p* < 0.10.

## 3. Results

### Galectin Expression in Normal Pregnancy

**Figure 1:**
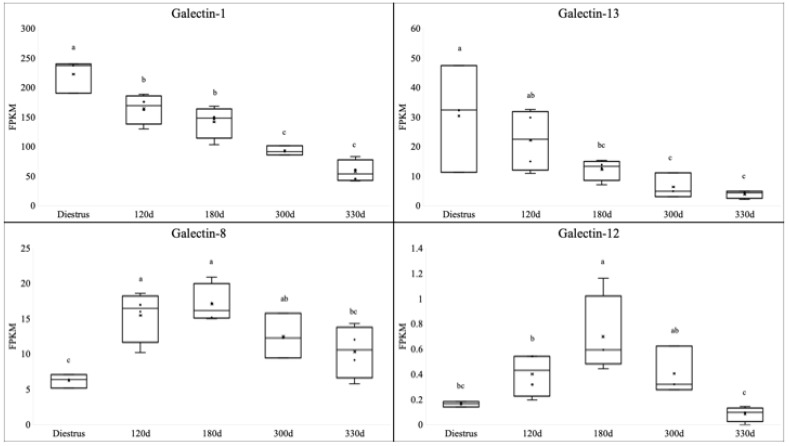
Expression of galectins in the endometrium during equine gestation. The expression of galectin-1 and galectin-13 followed a similar profile and were highest in the nonpregnant mare. This expression then decreased throughout gestation and was lowest in the 330 d gestation samples. A similar profile was noted for the expression of galectin-8 and galectin-12, where expression was low in the nonpregnant endometrium, increased in the pregnant endometrium, and reached the highest expression levels in mid-gestation at 180 d of gestation. The expression of galectin-8 and galectin-12 then decreased as parturition neared, and they were at nonpregnant levels by 330 d of gestation. All gene expression data are displayed as the fragments per kilobase of exon per million mapped (FPKM) ± SEM. ^a,b,c^ values with different superscripts differ; *p* < 0.05.

In the endometrium, galectin-1 (*p* < 0.01), galectin-8 (*p* < 0.01), galectin-12 (*p* < 0.01), and galectin-13 (*p* = 0.01) were altered during gestation (Figure 1). Two profiles could be noted for these galectins. Both galectin-1 and galectin-13 had the highest expression in the diestrus nonpregnant endometrium, and this then declined linearly towards parturition, with the lowest expression levels noted in the 330 d endometrium. In contrast, galectin-8 and galectin-12 were lowest in terms of expression in the diestrus endometrium. The highest expression levels were noted for both these galectins in the 120 d endometrium, followed by a linear decline towards parturition.

**Figure 2:**
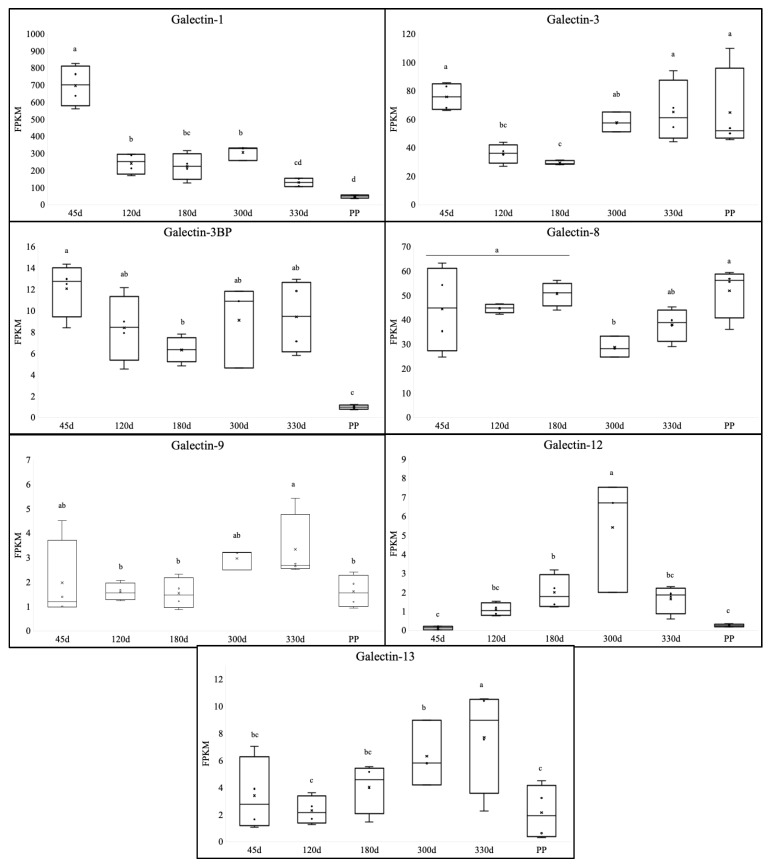
Expression of galectins in the chorioallantois during equine gestation. The expression of galectin-1 was highest in the 45 d chorioallantois and experienced a precipitous decline at 120 d, and expression levels remained low throughout the remainder of gestation, in addition to postpartum. Galectin-3 expression was also high in 45 d chorioallantois and experienced a decline in mid-gestation, but the expression levels of galectin-3 elevated during late-gestation and remained elevated postpartum. A similar profile was noted for galectin-3BP and galectin-9, where expression was high in 45 d chorioallantois, decreased in mid-gestation (120 d/180 d), increased in late-gestation (300 d/330 d), and had a precipitous decline in the postpartum chorioallantois. Galectin-8 remained in a relative steady-state throughout gestation, and only a decrease between 180 d and 300 d was noted. In contrast, galectin-12 expression was highest at 300 d of gestation, with decreased expression both in early- and mid-gestation, in addition to postpartum. All gene expression data are displayed as the fragments per kilobase of exon per million mapped (FPKM) ± SEM. ^a,b,c,d^ values with different superscripts differ; *p* < 0.05.

In the chorioallantois, galectin-1 (*p* < 0.001), galectin-3 (*p* = 0.006), galectin-3BP (*p* < 0.001), galectin-8 (*p* = 0.049), galectin-12 (*p* < 0.001), and galectin-13 (*p* = 0.03) were all significantly altered during gestation (Figure 2). Additionally, there was a trend towards an alteration in the expression of galectin-9 (*p* = 0.10). The expression of galectin-L/GRP (*p* = 0.12) and galectin-4 (*p* = 0.5) did not change during gestation, and the expression of galectin-7, galectin-9, and galectin-2 was consistently below detectable levels. Similar to within the endometrium, multiple galectins followed similar profiles throughout gestation. Both galectin-1 and galectin-3BP were higher in the chorioallantois during pregnancy compared to postpartum, with the highest expression levels noted at 45 d of gestation. Galectin-9 and galectin-13 also experienced a dramatic decrease in expression in the postpartum chorioallantois in comparison to the 330 d tissue, but this was to levels noted in early pregnancy (45 d/120 d). Both transcripts experienced the highest expression in late-gestation (300 d/330 d) before declining. Galectin-12 followed a distinct profile in comparison to the other galectins, with a precipitous increase from 45 d to 300 d, followed by a linear decline toward parturition. Postpartum galectin-12 was comparable to that of the 45 d pregnancy.

In contrast to the transcripts that decreased in the postpartum chorioallantois, galectin-3 and galectin-8 were found to have increased expression in both early pregnancy (45 d) and the postpartum chorion. Galectin-3 was found to decrease from 45 d of gestation to 180 d, but then the expression levels increased for the remaining gestation, while galectin-8 only decreased at 300 d of gestation but remained stable for the majority of timepoints assessed. Galectin-5, galectin-6, galectin-10, galectin-11, galectin-14, and galectin-15 were not found within the equine placenta.

## 4. Discussion

Galectins are multifunctional regulators of a variety of perturbations in the maternal environment during pregnancy that are of great interest in reproduction due to their high expression patterns at the maternal-fetal interface. In this study, we evaluated the expression of various galectins throughout normal equine gestation within the tissues making up the feto-maternal interface. The pathways through which these galectins act could not be confirmed within the confines of this study, but in other species evaluated, they have been found to act via both cytolosic and nuclear mechanisms to have downstream effects impacting inflammation, cell migration, and immune responses [23]. To our knowledge, we are the first to describe the galectinology of equine reproduction, a topic which deserves considerable future attention. 

Various profiles were noted within the galectin profiles of equine pregnancy, and this included within both the endometrium and chorioallantois. Galectin-1 and galectin-13 experienced similar profiles in both endometrium and chorioallantois, where expression was the highest in the nonpregnant endometrium and early-gestation chorioallantois, and this expression profile decreased as the gestational length increased. Galectin-1 is believed to be involved in implantation and angiogenesis in other species and has been identified in both endometrium and chorion in addition to circulation in the human and mouse [24,25]. In the human, galectin-1 expression is highly endocrine-controlled and maintained in both the 1st and 2nd trimester, following a comparable profile to progesterone at this time. This galectin has been found to regulate the progesterone output from granulosa cells in the human [26]. It appears that galectin-1 has a variety of receptors through which it enacts its downstream function, including integrin, actin, and various cell surface receptors, including CD4, CD45, and CD43 [27]. In the horse, galectin-1 expression was the highest at 45 d, further confirming its role in implantation, which has been previously described [16]. This then decreased dramatically by 120 d of gestation, which coincides with the decline in ovarian-produced progesterone within the equine species at this time [12]. In addition to its role in implantation, galectin-1 is involved in the induction of immunomodulatory Tregs [5], which have recently been described within the feto-maternal interface of the equine pregnancy, thereby indicating a potential positive regulator of the maturation of this subtype of lymphocytes [15]. Galectin-13 is also known as placenta protein 13 (PP13) and has been found primarily in the syncytiotrophoblast cells of the placenta in humans [28]. Galectin-13 is believed to dilate both uterine veins and arteries during human gestation due to its ability to increase prostaglandin signaling [29]. In humans, serum galectin-13 was found to increase as gestation progressed and is undetectable within 2–5 weeks postpartum [30]. A similar profile was noted in the horse, where the expression of galectin-13 in the chorioallantois increased as the gestational length progressed, reaching the highest expression levels at 11 months of gestation before declining dramatically in the postpartum chorioallantois. Galectin-13 is believed to be immunoregulatory in a variety of species and enacts this function through disabling macrophage function and diverting Tregs [31], although its function in the horse is unknown [31].

The second endometrial profile found both galectin-8 and galectin-12 to be the lowest in terms of expression in the diestrus endometrium, although the expression of these two galectins vastly differed in the pregnant chorioallantois. Galectin-8 is known as a “tandem repeat”-type galectin, therefore consisting of two CRDs linked by a peptide. This galectin is expressed highly in both the endometrium and chorion of early pregnancy in humans [7], and this expression pattern was also noted in the horse. In the endometrium, the lowest expression level was observed in the diestrus animal, and this increased dramatically at 120 d, followed by a linear decline towards parturition. In the chorioallantois, the highest expression was noted from 45 d to 180 d and again postpartum. This coincides greatly with the function of galectin-8, as the early-to-mid-gestation placenta experiences considerable angiogenesis and remodeling of the extracellular matrix [32,33]. Galectin-8 has two distinct effects on CD4+ T cells (Th1/Th2/Th17/Treg): high concentrations of galectin-8 induce antigen-independent proliferation, while low concentrations induce and stimulate antigen-dependent processes, and this is controlled by the tandem repeat aspect of the lectin [34]. Little is understood about the role of galectin-12 in pregnancy, although it is believed to primarily function in the turnover and maturation of adipocytes [35]. This galectin has been described to regulate the growth, differentiation, and lipolysis of these cells, in addition to modulating inflammation [36,37]. In the human, galectin-12 has been found to inhibit STAT3 signaling, leading to decreased pro-inflammatory signaling and the activation of cell survival outcomes [37]. In bovines, galectin-12 was found to be down-regulated in the pregnant animal in comparison to the cycling animal, although, to our knowledge, this has not been investigated in the human [38]. This is in disagreement with the findings in the horse, as galectin-12 was up-regulated in the pregnant mare, and the expression increased as the pregnancy progressed towards mid- to late-gestation. The physiology of this could not be determined under the confines of this study. 

A subset of the galectins investigated did not alter within the pregnant endometrium, while changes were noted in the chorioallantois, and this included galectin-3, galectin-3BP, and galectin-9, although no clear profile was noted. With regard to galectin-3, a heightened expression in both early- and late-gestation was observed in the pregnant mare, with a suppression at 120–180 d. This is in contrast to what has been reported in other species. In mice, the expression of galectin-3 is selectively upregulated in the endometrium of early pregnancy, and this is followed by a heightened expression in the chorioallantois of late gestation; this has also been shown in the human [24,39]. This difference in galectin-3 expression may be due to the variations in the placental physiology in the horse, but this could not be determined within the present study. Galectin-3 is involved in the transcription and translation of various proteins involved in many aspects of biology, including cell growth, apoptosis, differentiation, angiogenesis, fibrosis, and inflammation [40]. Expressed throughout the body, it is found within both innate and adaptive immune cells and can function as both pro- and anti-inflammatory [41,42]. In tumor cells, galectin-3 has been found to activate Ras/Raf/MEK/ERK signaling, leading to cell survival and pro-survival outcomes [43]. Considerably less is understood regarding the function of galectin-3BP in reproduction, although it has been identified in both colostrum and neonatal umbilical blood [44,45]. Galectin-3 is the receptor/ligand for galectin-3BP, and both function as secretory proteins. In the horse, the expression of galectin-3BP was altered throughout gestation in the chorioallantois, but not in the endometrium. Galectin-3BP expression remained elevated throughout pregnancy but was dramatically decreased in the postpartum chorioallantois. A similar decrease in the postpartum chorioallantois was noted for galectin-9, although the highest expression levels were observed in the prepartum sample (330 d). This is in contrast to other species, where galectin-9 is primarily expressed in the endometrium and, to a lesser extent, within the placenta, where the expression of galectin-9 is found to be highest in the first trimester. In the evaluated species, galectin-9 acts as an immunomodulator. Galectin-9 has been shown to stimulate the immune response by activating dendritic cells, but it primarily suppresses the immune response through increasing the apoptosis of Th1 cells [46] and promoting the maturation of FoxP3+ Treg cells [47,48]. The heightened galectin-9 expression noted in late equine gestation may therefore be connected to the heightened expression of Treg-related transcripts within the same tissue at this gestational age [15]. 

In conclusion, the expression of galectins at the maternal-fetal interface is both dynamic and dependent on the specific galectin in question. It is believed that galectins in the fetal membranes, including the chorioallantois, may regulate tissue development, promote antimicrobial effects, and participate in the regulation of inflammation, all of which may participate in the response to placental infection.

## Data Availability

The data presented in this study are available in NCBI Sequence Read Archive via the Gene Expression Omnibus (GEO), accession numbers GSE136691 and GSE108279.
